# Piperazin-1-ium tri­aqua­di­bromido­sodium

**DOI:** 10.1107/S241431462500985X

**Published:** 2025-11-11

**Authors:** William T. A. Harrison

**Affiliations:** a Department of Chemistry, University of Aberdeen, Meston Walk, Aberdeen AB24 3UE, Scotland, United Kingdom; Purdue University, USA

**Keywords:** crystal structure, complex ion, sodium, trigonal bipyramid

## Abstract

In the title compound, (C_4_H_11_N_2_)[NaBr_2_(H_2_O)_3_], the complete organic cation, which adopts a typical chair conformation, is generated by crystallographic inversion symmetry and one of the N-bonded H atoms is half occupied. The sodium ion (site symmetry *m*) at the centre of the complex anion adopts a distorted trigonal–bipyramidal coordination geometry with the water mol­ecules in the equatorial sites and the bromide ions in the axial sites. In the extended structure, O—H⋯Br hydrogen bonds generate a porous ‘honeycomb’ three-dimensional network of complex anions encapsulating [010] channels occupied by the cations, which are linked to each other by N—H⋯N hydrogen bonds and anchored to the honeycomb network *via* N—H⋯Br hydrogen bonds.

## Abstract 2

In the title compound, the complete organic cation, which adopts a typical chair conformation, is generated by crystallographic inversion symmetry and one of the N-bonded H atoms is half occupied. The sodium ion (site symmetry *m*) at the centre of the complex anion adopts a distorted trigonal–bipyramidal coordination geometry with the water mol­ecules in the equatorial sites and the bromide ions in the axial sites. In the extended structure, O—H⋯Br hydrogen bonds generate a porous ‘honeycomb’ three-dimensional network of complex anions encapsulating [010] channels occupied by the cations.

## Graphical abstract

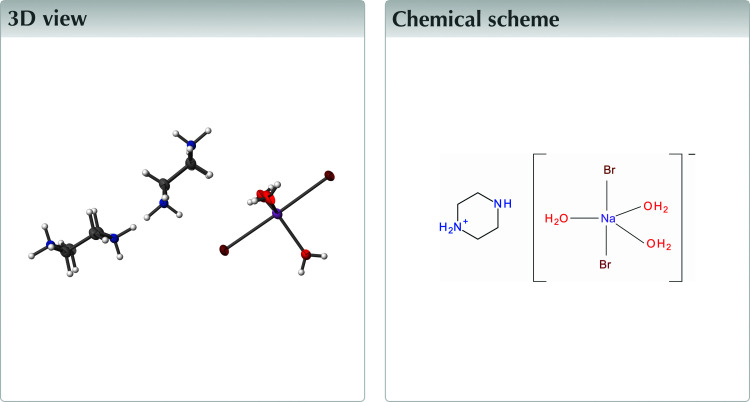

## Structure description

Some time ago we reported a family of ‘hybrid’ organic/inorganic perovskites of general formulae *RAX*_3_ and *RAX*_3_·H_2_O where *R* is a doubly protonated organic dication such as piperazinium (piperazin-1,4-diium) (C_4_H_12_N_2_^2+^) or ‘dabconium’ (1,4-diazo­niabi­cyclo­[2.2.2]octa­ne) (C_6_H_14_N_2_^2+^), *A* is an alkali metal (K^+^, Rb^+^, Cs^+^) and *X* is a halide ion (Cl^−^, Br^−^) (Paton & Harrison, 2010 ▸). These phases consist of a three-dimensional network of corner-sharing *AX*_6_ octa­hedra analogous to the metal–oxide octa­hedral framework in inorganic perovskites (Tilley, 2016 ▸) with the lacunae occupied by the organic cations, and in some cases, also by water mol­ecules. Other workers (Zhang *et al.*, 2017 ▸; Pan *et al.*, 2017 ▸; Chen *et al.*, 2018 ▸) have substanti­ally expanded this family and shown that some of these phases exhibit striking ferroelectric behaviour akin to that shown by classical oxide perovskites. We later prepared the ‘missing link’ hemihydrate *RA*Br_3_·0.5H_2_O hybrid perovskites (Ferrandin *et al.*, 2019 ▸) where *R* is the 1-methyl­piperizine-1,4-diium cation (C_5_H_14_N_2_^2+^) and *A* = K^+^, Rb^+^ or Cs^+^: the known *RAX*_3_ and *RAX*_3_·H_2_O hybrid perovskites were surveyed in this paper. In an attempt to prepare a new hybrid perovskite of putative formula C_4_H_12_N_2_·NaBr_3_·*x*H_2_O we reacted piperazine and acidified sodium bromide in water but instead, the unexpected title compound, (C_4_H_11_N_2_)^+^[NaBr_2_(H_2_O)_3_]^−^ (**I**), arose and we now describe its structure.

**Figure 1:**
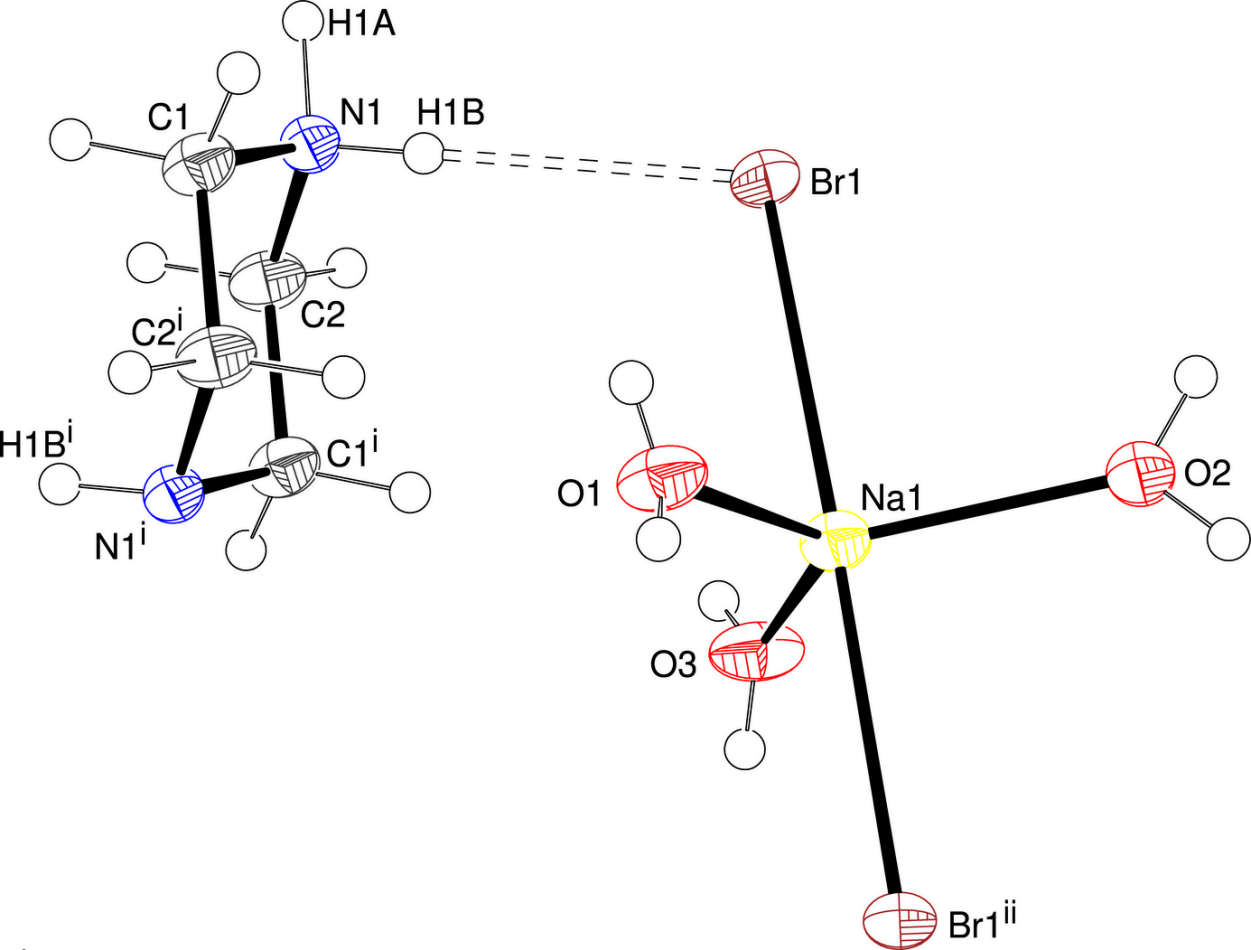
The asymmetric unit of (**I**) expanded to show the complete cation and complex anion showing 50% displacement ellipsoids. The hydrogen bond is shown as a double-dashed line. Symmetry codes: (i) −*x*, 1 − *y*, 2 − *z*; (ii) *x*, 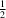 − *y*, *z*. Atom H1*A* is statistically disordered and is shown in just one location.

The asymmetric unit of (**I**) (Fig. 1 ▸), which crystallizes in the ortho­rhom­bic space group *Pnma*, consists of two methyl­ene groups, one NH_1.5_ grouping (the H-atom disorder is described below), one Na^+^ ion (site symmetry *m*), three water mol­ecules (O site symmetries *m*) and one bromide ion. Crystal symmetry (an inversion centre at 0, 1/2, 1 for the asymmetric atoms) generates the complete C_4_H_11_N_2_^+^ piperazin-1-ium cation, which adopts a normal chair conformation (Dennington & Weller, 2018 ▸) with the N atoms displaced by ±0.631 (6) Å from the plane of the four C atoms. Atom H1*A*, which has an equatorial orientation with respect to the chair, must be 1/2 occupied, otherwise a chemically unreasonable H1*A*⋯H1*A*^i^ [symmetry code: (i) *x*, 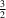 − *y*, *z*] short contact of ∼0.76 Å would arise. This overall mono-protonation of the organic species leads to a disordered N1—H1*A*⋯N1/N1⋯H1*A*—N1 hydrogen bond in the extended structure of (**I**) (see below) and (of course) establishes proper charge balance with the complex anion.

The complete [NaBr_2_(H_2_O)_3_]^−^ complex anion in (**I**) is generated by a mirror plane at *y* = 1/4 for the asymmetric atoms. This results in an unusual distorted trigonal pyramidal coordination geometry for the sodium ion with the O atoms (mean Na—O = 2.272 Å) occupying the equatorial sites and the bromide ions the axial sites. The Br1—Na1—Br1^ii^ [symmetry code: (ii) *x*, 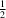 − *y*, *z*] moiety is almost linear at 178.38 (8)° while the O—Na—O bond angles (Table 1 ▸) show some deviations from ideal local *D*_3*h*_ symmetry with the minimum and maximum angles being 113.36 (18) and 131.29 (19)°, respectively: the τ_5_ parameter (Addison *et al.*, 1984 ▸) is 0.78 compared to 1.00 for a regular trigonal-prismatic geometry. The sodium bond-valence sum (BVS) of 1.25 valence units (expected value 1.00 v.u.) using the BVS data collated by Brown (2020 ▸), suggests a degree of ‘overbonding’ for the metal ion in (**I**).

**Figure 2:**
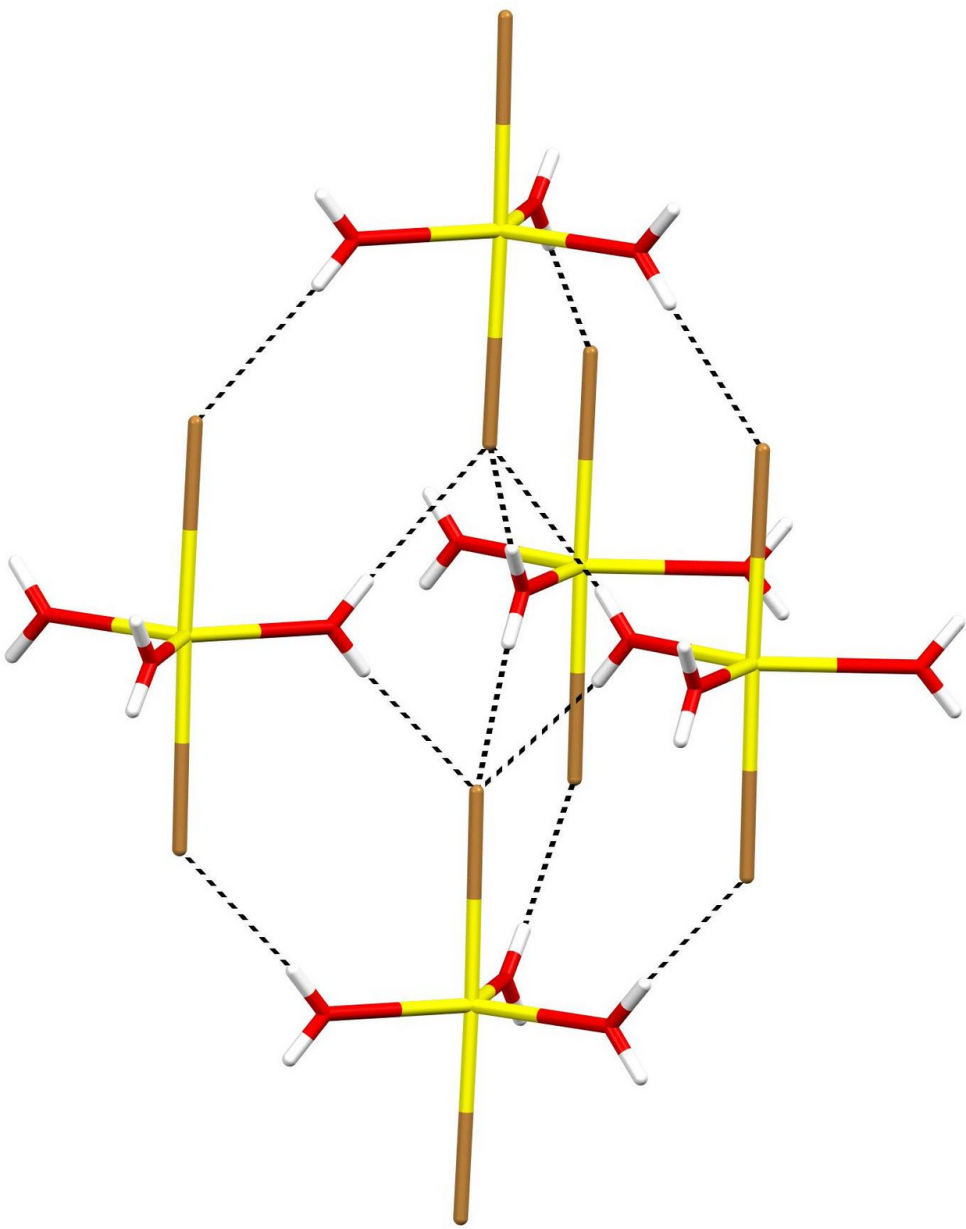
Fragment of the extended structure of (**I**) showing a network of O—H⋯Br hydrogen bonds (black dashed lines) in which each bromide ion accepts three such bonds. The Br^−^ ion also accepts an N—H⋯Br hydrogen bond from the organic cation (not shown).

**Figure 3:**
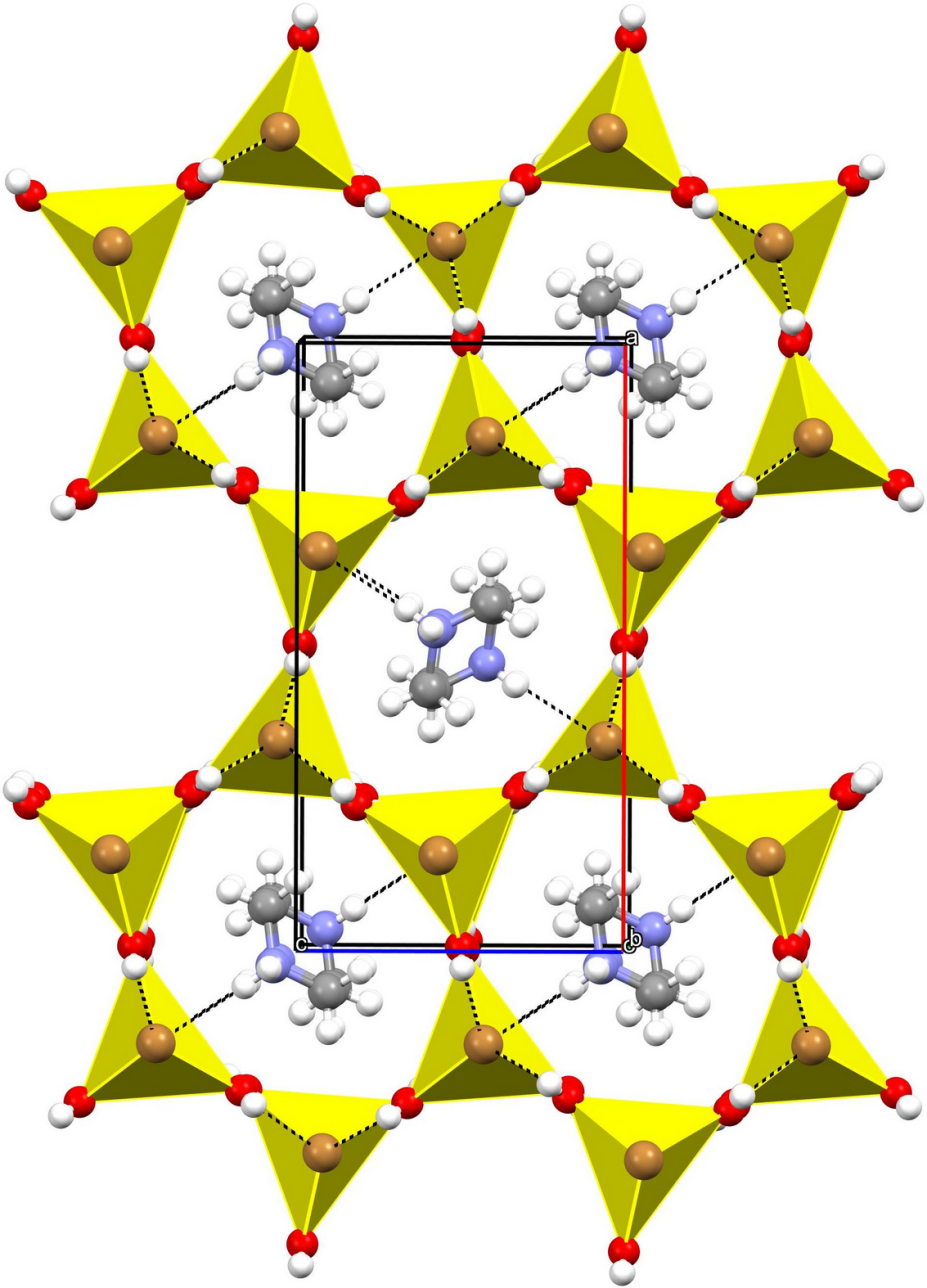
The unit-cell packing in (**I**) viewed down [010] with the cations shown in ball-and-stick representation and the complex anions in polyhedral representation. Hydrogen bonds are shown as black dashed lines.

In the extended structure of (**I**), the complex anions are linked by O—H⋯Br hydrogen bonds (Table 2 ▸). All six water H atoms (three being symmetry generated by the mirror plane) participate in these links. Each water mol­ecule forms a hydrogen bond to an adjacent complex anion both ‘above’ (with respect to the *b*-axis direction) and below it and each bromide ion accepts three such bonds (Fig. 2 ▸). Given their H⋯Br lengths and near-linear bond angles, they may be regarded as strong hydrogen bonds. Collectively, these hydrogen bonds result in a three-dimensional ‘honeycomb’ network encapsulating [010] channels occupied by the organic cations (Fig. 3 ▸). As noted above, the cations are linked by disordered N1—H1*A*⋯N1 hydrogen bonds into [010] chains and finally, N1—H1*B*⋯Br1 hydrogen bonds help to anchor the cations in the [010] channels with respect to the honeycomb framework.

A survey of the Cambridge Structural Database (Groom *et al.*, 2016 ▸; updated to October 2025) did not yield any matches for the complex anion reported here. As to why the intended compound did not form, we may speculate that the sodium cation (ionic radius for Na^+^ = 1.02 Å compared to 1.38 Å for K^+^) is too small to permit the formation of a perovskite-like network of corner-sharing NaBr_6_ octa­hedra in a hybrid perovskite. However, it should be noted that sodium bromide is a very well-known phase that contains NaBr_6_ octa­hedra in which the Na—Br separation is about 2.987 Å (Nickels *et al.*, 1949 ▸) and it may be the case that we simply failed to find the right synthetic conditions to make the target hybrid perovskite.

## Synthesis and crystallization

Compound (**I**) was prepared by mixing 0.43 g of C_4_H_10_N_2_, 0.51 g of NaBr, 10 ml of 1.0 *M* HBr solution and 20 ml of water (piperazine:Na:Br molar ratio ≃ 1:1:3), which resulted in a colourless solution. The solution was left in a Petri dish at room temperature and blade-like colourless crystals of (**I**) formed as the water evaporated over a few days. Mixtures with less added acid led to recrystallized KBr and with more acid produced the known phase (C_4_H_12_N_2_)Br_2_·H_2_O (Bujak, 2015 ▸).

## Refinement

Crystal data, data collection and structure refinement details are summarized in Table 3 ▸. The O– and N-bound H atoms were located in difference maps and their positions were freely refined. The C-bound H atoms were located geometrically (C—H = 0.98 Å) and refined as riding atoms. The constraint *U*_iso_(H) = 1.2*U*_eq_(carrier) was applied in all cases. Atom H1*A* is disordered by symmetry about a crystallographic mirror plane: lower-symmetry space groups were investigated to see if an ordered model could be developed but these did not resolve the disorder and the refinements showed excessive correlation between parameters and unrealistic displacement ellipsoids, which are signs that the symmetry is too low, so space group *Pnma* was assumed.

## Supplementary Material

Crystal structure: contains datablock(s) I, global. DOI: 10.1107/S241431462500985X/zl4089sup1.cif

Structure factors: contains datablock(s) I. DOI: 10.1107/S241431462500985X/zl4089Isup2.hkl

CCDC reference: 2500621

Additional supporting information:  crystallographic information; 3D view; checkCIF report

## Figures and Tables

**Table 1:** Selected geometric parameters (Å, °)

Na1—O1	2.266 (5)	Na1—O2	2.276 (4)
Na1—O3	2.274 (4)	Na1—Br1	2.9160 (4)
			
O1—Na1—O3	131.29 (19)	O3—Na1—Br1	89.37 (4)
O1—Na1—O2	113.36 (18)	O2—Na1—Br1	90.73 (4)
O3—Na1—O2	115.36 (19)	Br1—Na1—Br1^i^	178.38 (8)
O1—Na1—Br1	90.03 (4)		

Symmetry code: (i) 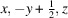.

**Table 2:** Hydrogen-bond geometry (Å, °)

*D*—H⋯*A*	*D*—H	H⋯*A*	*D*⋯*A*	*D*—H⋯*A*
O1—H1⋯Br1^ii^	0.75 (4)	2.65 (4)	3.386 (3)	167 (5)
O2—H2⋯Br1^iii^	0.84 (4)	2.52 (4)	3.352 (3)	172 (4)
O3—H3⋯Br1^iv^	0.78 (4)	2.59 (5)	3.365 (3)	170 (5)
N1—H1*A*⋯N1^v^	1.01 (7)	1.77 (7)	2.773 (6)	172 (6)
N1—H1*B*⋯Br1	0.88 (4)	2.59 (4)	3.467 (3)	177 (4)

Symmetry codes: (ii) 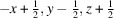; (iii) 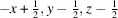; (iv) 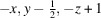; (v) 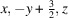.

**Table 3:** Experimental details

Crystal data	
Chemical formula	(C_4_H_11_N_2_)[NaBr_2_(H_2_O)_3_]
*M*_r_	324.01
Crystal system, space group	Orthorhombic, *Pnma*
Temperature (K)	120
*a*, *b*, *c* (Å)	14.2622 (12), 10.6066 (8), 7.6876 (6)
*V* (Å^3^)	1162.93 (16)
*Z*	4
Radiation type	Mo *K*α
μ (mm^−1^)	6.99
Crystal size (mm)	0.48 × 0.26 × 0.07

Data collection	
Diffractometer	Rigaku R-AXIS CCD
Absorption correction	Multi-scan (*CrystalClear*; Rigaku, 2014 ▸)
*T*_min_, *T*_max_	0.400, 1.000
No. of measured, independent and observed [*I* > 2σ(*I*)] reflections	14657, 1400, 1324
*R*_int_	0.100
(sin θ/λ)_max_ (Å^−1^)	0.650

Refinement	
*R*[*F*^2^ > 2σ(*F*^2^)], *wR*(*F*^2^), *S*	0.045, 0.094, 1.27
No. of reflections	1400
No. of parameters	76
H-atom treatment	H atoms treated by a mixture of independent and constrained refinement
Δρ_max_, Δρ_min_ (e Å^−3^)	0.67, −1.14

Computer programs: *CrystalClear* (Rigaku, 2014 ▸), *SHELXT* (Sheldrick, 2015*a* ▸), *SHELXL2019/2* (Sheldrick, 2015*b* ▸), *ORTEP-3 for Windows* (Farrugia, 2012 ▸) and *publCIF* (Westrip, 2010 ▸).

## full crystallographic data

### Piperazin-1-ium triaquadibromidosodium Crystal data

**Table:**

(C_4_H_11_N_2_)[NaBr_2_(H_2_O)_3_]	*D*_x_ = 1.851 Mg m^−^^3^
*M**_r_* = 324.01	Mo *K*α radiation, λ = 0.71073 Å
Orthorhombic, *P**n**m**a*	Cell parameters from 6537 reflections
*a* = 14.2622 (12) Å	θ = 2.4–27.5°
*b* = 10.6066 (8) Å	µ = 6.99 mm^−^^1^
*c* = 7.6876 (6) Å	*T* = 120 K
*V* = 1162.93 (16) Å^3^	Blade, colourless
*Z* = 4	0.48 × 0.26 × 0.07 mm
*F*(000) = 640	

### Piperazin-1-ium triaquadibromidosodium Data collection

**Table:**

Rigaku R-AXIS CCD diffractometer	1324 reflections with *I* > 2σ(*I*)
ω scans	*R*_int_ = 0.100
Absorption correction: multi-scan (CrystalClear; Rigaku, 2014)	θ_max_ = 27.5°, θ_min_ = 2.9°
*T*_min_ = 0.400, *T*_max_ = 1.000	*h* = −18→17
14657 measured reflections	*k* = −13→13
1400 independent reflections	*l* = −9→10

### Piperazin-1-ium triaquadibromidosodium Refinement

**Table:**

Refinement on *F*^2^	Primary atom site location: dual
Least-squares matrix: full	Hydrogen site location: mixed
*R*[*F*^2^ > 2σ(*F*^2^)] = 0.045	H atoms treated by a mixture of independent and constrained refinement
*wR*(*F*^2^) = 0.094	*w* = 1/[σ^2^(*F*_o_^2^) + (0.0241*P*)^2^ + 2.2228*P*] where *P* = (*F*_o_^2^ + 2*F*_c_^2^)/3
*S* = 1.27	(Δ/σ)_max_ = 0.001
1400 reflections	Δρ_max_ = 0.67 e Å^−^^3^
76 parameters	Δρ_min_ = −1.14 e Å^−^^3^
0 restraints	

### Piperazin-1-ium triaquadibromidosodium Special details

**Table:**

Geometry. All esds (except the esd in the dihedral angle between two l.s. planes) are estimated using the full covariance matrix. The cell esds are taken into account individually in the estimation of esds in distances, angles and torsion angles; correlations between esds in cell parameters are only used when they are defined by crystal symmetry. An approximate (isotropic) treatment of cell esds is used for estimating esds involving l.s. planes.

### Piperazin-1-ium triaquadibromidosodium Fractional atomic coordinates and isotropic or equivalent isotropic displacement parameters (Å^2^)

**Table:**

	*x*	*y*	*z*	*U*_iso_*/*U*_eq_	Occ. (<1)
Na1	0.16043 (13)	0.250000	0.5630 (3)	0.0249 (5)	
Br1	0.15785 (2)	0.52490 (3)	0.56542 (5)	0.02552 (16)	
O1	0.2377 (3)	0.250000	0.8205 (6)	0.0324 (9)	
H1	0.261 (3)	0.193 (4)	0.860 (6)	0.039*	
O2	0.2576 (3)	0.250000	0.3282 (5)	0.0318 (9)	
H2	0.280 (3)	0.190 (4)	0.272 (6)	0.038*	
O3	0.0046 (3)	0.250000	0.5006 (7)	0.0349 (9)	
H3	−0.030 (3)	0.193 (4)	0.496 (6)	0.042*	
N1	0.0284 (2)	0.6193 (3)	0.9243 (4)	0.0225 (6)	
H1A	0.032 (5)	0.714 (7)	0.914 (9)	0.027*	0.5
H1B	0.063 (3)	0.594 (4)	0.836 (6)	0.027*	
C1	0.0716 (3)	0.5725 (3)	1.0874 (5)	0.0264 (8)	
H1C	0.038009	0.608773	1.188345	0.032*	
H1D	0.137721	0.600804	1.093083	0.032*	
C2	−0.0681 (3)	0.5709 (3)	0.9025 (5)	0.0273 (8)	
H2A	−0.092890	0.598665	0.788475	0.033*	
H2B	−0.108701	0.606976	0.994359	0.033*	

### Piperazin-1-ium triaquadibromidosodium Atomic displacement parameters (Å^2^)

**Table:**

	*U*^11^	*U*^22^	*U*^33^	*U*^12^	*U*^13^	*U*^23^
Na1	0.0270 (11)	0.0202 (10)	0.0274 (12)	0.000	−0.0015 (8)	0.000
Br1	0.0305 (3)	0.0167 (2)	0.0294 (3)	0.00086 (12)	0.00097 (13)	−0.00139 (12)
O1	0.040 (2)	0.0202 (17)	0.037 (2)	0.000	−0.0077 (19)	0.000
O2	0.041 (2)	0.0203 (17)	0.034 (2)	0.000	0.0109 (18)	0.000
O3	0.027 (2)	0.0203 (17)	0.057 (3)	0.000	−0.0062 (19)	0.000
N1	0.0259 (15)	0.0177 (13)	0.0241 (16)	0.0009 (11)	0.0034 (12)	−0.0004 (11)
C1	0.0308 (19)	0.0205 (17)	0.028 (2)	0.0008 (14)	−0.0043 (15)	−0.0015 (14)
C2	0.0278 (18)	0.0205 (17)	0.034 (2)	0.0018 (14)	−0.0059 (15)	−0.0001 (14)

### Piperazin-1-ium triaquadibromidosodium Geometric parameters (Å, º)

**Table:**

Na1—O1	2.266 (5)	O3—H3^i^	0.78 (4)
Na1—O3	2.274 (4)	N1—C2	1.479 (5)
Na1—O2	2.276 (4)	N1—C1	1.482 (4)
Na1—Br1	2.9160 (4)	N1—H1A	1.01 (7)
Na1—Br1^i^	2.9161 (4)	N1—H1B	0.88 (4)
O1—H1	0.75 (4)	C1—C2^ii^	1.524 (5)
O1—H1^i^	0.75 (4)	C1—H1C	0.9900
O2—H2	0.84 (4)	C1—H1D	0.9900
O2—H2^i^	0.84 (4)	C2—H2A	0.9900
O3—H3	0.78 (4)	C2—H2B	0.9900
			
O1—Na1—O3	131.29 (19)	C2—N1—C1	111.5 (3)
O1—Na1—O2	113.36 (18)	C2—N1—H1A	112 (4)
O3—Na1—O2	115.36 (19)	C1—N1—H1A	112 (4)
O1—Na1—Br1	90.03 (4)	C2—N1—H1B	109 (3)
O3—Na1—Br1	89.37 (4)	C1—N1—H1B	108 (3)
O2—Na1—Br1	90.73 (4)	H1A—N1—H1B	102 (5)
O1—Na1—Br1^i^	90.03 (4)	N1—C1—C2^ii^	111.3 (3)
O3—Na1—Br1^i^	89.37 (4)	N1—C1—H1C	109.4
O2—Na1—Br1^i^	90.73 (4)	C2^ii^—C1—H1C	109.4
Br1—Na1—Br1^i^	178.38 (8)	N1—C1—H1D	109.4
Na1—O1—H1	124 (4)	C2^ii^—C1—H1D	109.4
Na1—O1—H1^i^	124 (4)	H1C—C1—H1D	108.0
H1—O1—H1^i^	107 (7)	N1—C2—C1^ii^	111.8 (3)
Na1—O2—H2	130 (3)	N1—C2—H2A	109.3
Na1—O2—H2^i^	130 (3)	C1^ii^—C2—H2A	109.3
H2—O2—H2^i^	100 (6)	N1—C2—H2B	109.3
Na1—O3—H3	129 (3)	C1^ii^—C2—H2B	109.3
Na1—O3—H3^i^	129 (3)	H2A—C2—H2B	107.9
H3—O3—H3^i^	101 (7)		
			
C2—N1—C1—C2^ii^	54.4 (4)	C1—N1—C2—C1^ii^	−54.7 (4)

Symmetry codes: (i) *x*, −*y*+1/2, *z*; (ii) −*x*, −*y*+1, −*z*+2.

### Piperazin-1-ium triaquadibromidosodium Hydrogen-bond geometry (Å, º)

**Table:**

*D*—H···*A*	*D*—H	H···*A*	*D*···*A*	*D*—H···*A*
O1—H1···Br1^iii^	0.75 (4)	2.65 (4)	3.386 (3)	167 (5)
O2—H2···Br1^iv^	0.84 (4)	2.52 (4)	3.352 (3)	172 (4)
O3—H3···Br1^v^	0.78 (4)	2.59 (5)	3.365 (3)	170 (5)
N1—H1*A*···N1^vi^	1.01 (7)	1.77 (7)	2.773 (6)	172 (6)
N1—H1*B*···Br1	0.88 (4)	2.59 (4)	3.467 (3)	177 (4)

Symmetry codes: (iii) −*x*+1/2, *y*−1/2, *z*+1/2; (iv) −*x*+1/2, *y*−1/2, *z*−1/2; (v) −*x*, *y*−1/2, −*z*+1; (vi) *x*, −*y*+3/2, *z*.
